# Comparative Analysis of the Structure, Properties and Internal Stresses of MAG Welded Joints Made of S960QL Steel Subjected to Heat Treatment and Pneumatic Needle Peening

**DOI:** 10.3390/ma18235363

**Published:** 2025-11-28

**Authors:** Jacek Górka, Mateusz Przybyła, Bernard Wyględacz

**Affiliations:** 1 Department of Welding Engineering, Silesian University of Technology, Konarskiego Str. 18a, 44-100 Gliwice, Poland; 2 FAMET S.A., Szkolna Str. 15, 47-225 Kędzierzyn Koźle, Poland; mateusz.przybyla@famet.com.pl

**Keywords:** S960QL steel, MAG welding, welding technology qualification, peening, internal stresses

## Abstract

The aim of the research was to analyse the impact of peening each of the beads on the properties of a butt joint made of S960QL steel welded with ceramic backing on a robotic workstation using the 135 (MAG) method, and to determine the impact of pneumatic needle peening on the stress level. This analysis was based on a comparison of three butt joints: in the as-welded state, with each weld bead peened and post-weld heat treatment—stress relief annealing—performed. High-frequency peening (90 Hz) of each weld was performed to reduce stresses in the welded joint by introducing tensile stresses into it. A Weld Line 10 pneumatic hammer from PITEC GmBH was used for this purpose. The test joints obtained were tested in accordance with the requirements of EN ISO 15614-1. In order to determine the state of residual stresses, stress measurements were carried out using the Barkhausen effect based on the testing procedure of the technology supplier, NNT. This meter measures the intensity of the Barkhausen effect using a standard probe (with a single core). In order to verify the stress measurement using the Barkhausen method, stress measurements were performed using the XRD sin 2ψ technique based on the X’Pert Stress Plus program, which contains a database of material constants necessary for calculations. Structural studies, including phase analysis and crystallographic grain orientation, were performed using the backscattered electron diffraction method with a high-resolution scanning electron microscope and an EBSD (Electron Backscatter Diffraction) detector, as well as EDAX OIM analysis software. In addition, X-ray diffraction testing was performed on a Panalytical X’Pert PRO device using filtered cobalt anode tube radiation (λ = 1.79021 A). Qualitative X-ray phase analysis of the tested materials was performed in a Bragg–Brentano system using an Xcelerator strip detector. The tests showed that the high-frequency peening of each bead did not cause negative results in the required tests during qualification of the S960QL plate-welding technology compared to the test plates in the as-welded and post-stress-relief heat treatment states. Interpass peening of the weld face and HAZ resulted in a reduction in residual stresses after welding at a distance of 15 mm from the joint axis compared to the stress measurement result for the sample in the as-welded condition. This allows for a positive assessment of peening in terms of reducing the crack initiator in the form of the concentration of tensile stresses in the area of the fusion line and HAZ.

## 1. Introduction

The phenomena that accompany welding processes and are the cause of the most undesirable effects of welding include: volume changes associated with phase transformations; uneven and rapid heating and cooling; and changes in properties such as the Young’s modulus, E, yield strength, Re, or thermal expansion coefficient, α, during heating and cooling [1]. The most undesirable phenomena associated with the welding process are stresses and deformations in welded joints. Welding stress and deformation are difficult to control and manage simultaneously. Stresses pose a threat to the entire structure as they can potentially lead to its damage. Stresses result from the contraction of the weld during its cooling from the yield temperature, approximately 600–650 °C, to the ambient temperature at which the structure will be used. Welding processes invariably cause significant stresses due to the difference in thermal expansion and contraction of the weld caused by local heating of the joined elements. The magnitude of these stresses can reach the plasticity limit of the welded material. Welding stresses can cause significant deformation of structures and, in some cases, lead to their failure. Therefore, materials intended for welded structures should have sufficiently high plasticity [2]. It is worth noting that welding stresses arise not only in complex structures, but also in simple configurations, such as two freely lying plates. The issue of welding stresses is therefore of great importance, but is often underestimated and not taken into account in structural strength calculations [3,4,5]. Stresses that arise in the material as a result of mechanical or thermal processing, such as casting, rolling, hardening, forging or welding processes, and those that remain in the material after the process is complete, are called residual stresses [6]. Residual stresses are a type of internal stresses. Internal stresses are defined as stresses that balance each other out in a specific area without any external load. Due to the complex process of their formation, they are very difficult to predict and difficult to eliminate completely. Therefore, there is a need to search for and develop new technologies or alternative applications of existing techniques to reduce welding stresses and deformations. Heat treatment is a commonly used method of stress and strain relaxation in heavy industry. One alternative to classic post-welding heat treatment is peening. This method involves inter-pass peening or only treating the face of butt or fillet welds in order to introduce compressive stresses through plastic deformation. The advantage of this method is the possibility of performing both local and global, effective post-weld treatment [6,7,8]. Peening can be carried out using several systems, including conventional methods such as peening with an electric or pneumatic hammer. Among the relatively new methods of relaxation through High Frequency Mechanical Impact (HFMI), the following methods can be distinguished: Ultrasonic Peening Treatment (UPT), High Frequency Impact Treatment (HiFIT), Ultrasonic Peening (UP), Pneumatic Impact Treatment (PIT) and Ultrasonic Needle Peening (UNP). Each of these methods has been developed primarily to increase the effectiveness of the impact, the precision of the treatment and the comfort of work by reducing the impact on the personnel performing the treatment [8,9,10,11,12,13,14,15,16,17]. European standards do not determine whether the use of this method is a variable relevant to the welding process, hence the need to conduct research on key issues when qualifying arc welding technologies for metals, such as compliance with the requirements of PN-EN ISO 15614-1 and the impact of peening on the results of the required tests [18,19,20]. The current state of knowledge in the field of high-frequency re-peening or ultrasonic re-peening is extensive, but it should be noted here that most of the articles and studies in this area focus on improving fatigue strength through the use of HFMI technology; the guidelines of the International Institute of Welding [12,13,14,15] are an example of this. Therefore, the development of interpass and post-weld peening technologies will determine the more widespread and broader application of this approach to reduce stresses and deformations, especially when the established methods described above fail or are not practical due to material or technological limitations [20,21,22,23].

## 2. Methods and Materials

The aim of the research was to analyse the impact of the peening process on each bead on the properties of a butt joint welded with a ceramic backing on a robotic workstation using the 135 (MAG) method, and to determine the impact of peening on the level of residual stresses. This analysis was based on a comparison of three butt joints made of S960QL plate (Table 1), in the as-welded condition, with each weld bead peened and post-weld heat treatment—stress relief annealing—performed. The filler material used for welding was ED-FK 1000 wire from Fliess with a diameter of Ø1.2 mm (Table 2). Welding tests were in accordance with previously developed pWPSs at FAMET S.A.

### 2.1. Welding of the Test Plates

As part of the work, 3 V bevelled butt welded joints were fabricated. A ceramic pad was used to ensure proper penetration and formation of the weld root in the single-sided welding process. The welding process was carried out on a robotic station to ensure comparable welding parameters for each of the test plates. The shielding gas used was a 92% Ar + 8% CO_2_ EN ISO 14175 [24] M20 mixture. Table 3 shows the welding parameters for each of the test plate beads. The interpass temperature was the surface temperature measured before depositing the corresponding bead. The linear energy was a measure of the energy used to deposit a length unit of weld. The linear energy range results achieved show that welding on a robotic station allowed for almost identical welding conditions. The only difference seen in the sample peened was in terms of the interpass temperature, which was lower compared to the other test plates, due to the increased time between welding individual beads and the resultative cooling during peening.

### 2.2. Interpass Peening

High-frequency peening (90 Hz) of each bead was performed to reduce stress in the welded joint by introducing compressive stress. A Weld Line 10 pneumatic hammer from PITEC GmBH, Duisburg, Germany was used for this purpose. For the device to work effectively, the compressed air system must be pressurised to between 4 and 7 bar. During the peening process, the pressure was approximately 6.5 bar. Correct peening was achieved when all of the bead surface was treated with an 80 mm long mandrel and a tip radius of R = 2 mm, and the treatment itself was carried out manually at a speed of 20 cm/min.

### 2.3. Post-Weld Stress Relief Heat Treatment

The last test plate after welding was subjected to post-welding heat treatment, which is commonly used to reduce stresses and deformations resulting from welding processes. The heat treatment parameters were as follows: controlled heating (100 °C/h), soaking (temperature 580 °C) and controlled cooling (100 °C/h).

### 2.4. Acceptance Tests in Accordance with PN-EN ISO 15614-1:2017 [20]

In accordance with the requirements of the specification and qualification of metal welding technology, the test joints obtained were subjected to the following non-destructive tests:

- Visual (VT) in accordance with PN-EN ISO 17637:2023 [25];
- Penetration (PT) in accordance with PN-EN ISO 3452-1:2021 [26];
- Radiographic (RT) in accordance with PN-EN ISO 17636-1:2023 [27].

All non-destructive tests on the welded joints were successful, with the joints achieving quality level B.

The next step in the acceptance procedure is to carry out destructive tests:

- Tensile test in accordance with PN-EN ISO 4136:2022 [28];
- Transverse bending test in accordance with PN-EN ISO 5173:2023 [29];
- Impact test in accordance with PN-EN ISO 9016:2022 [30];
- Hardness test in accordance with PN-EN ISO 9015-1:2011 [31];
- Macroscopic test in accordance with PN-EN ISO 17639:2022 [32].

The impact tests were performed on undersized samples. For the transverse bending test, two samples for face bending and two samples for root bending were made.

### 2.5. Microstructure Analysis Using Electron Backscatter Diffraction

Structural studies, including phase analysis and crystallographic grain orientation, were performed using the backscattered electron diffraction method with a high-resolution Supra 35 scanning electron microscope from Zeiss, Oberkochen, Germany, an EBSD (Electron Backscatter Diffraction) detector from Zeiss, Oberkochen, Germany, and OIM v8.5 Analysis software from EDAX. EBSD tests were performed on metallographic sections with a step of 0.3 µm at 2000× magnification, performing analyses in two areas: in the native material area and in the HAZ for both the as-welded and high-frequency peened samples. The accelerating voltage was 20 kV and the working distance was 17 mm [33].

### 2.6. Stress Measurement Using Barkhausen Effect

Stress measurements were performed based on the testing procedure of the MagStress5c NNT Sp. z o.o., Gdańsk, Poland device supplier. The stress state was determined using a MagStress5c meter and the developed PB01 testing procedure, which describes how to determine the stress state using the Barkhausen effect (BE). This meter measured the intensity of BE using a standard single-core probe [34,35,36]. The direction of magnetisation thus determined the direction of the BE intensity test. The recorded BE voltage signal is converted in the meter into a BE intensity descriptor, which is the integral of the Barkhausen noise signal (INT) parameter, a measure of the effective voltage of the BE voltage signal. BE intensity measurements were collected and the INT value was determined on the face side in a direction parallel to the weld axis (R direction) for a series of measurement points. The MagStress version 5c system automatically converts the INT value into eps deformation according to the calibration function table stored in its memory, eps = F(X), where X is the quotient of the measured INT intensity and the INTref intensity value, which is assumed to be representative of the undeformed state (eps = 0). The meter records the INT value and the Si value, i.e., the stress, sigma, which is calculated by the rule sigma = E × eps, where E is Young’s modulus. The meter stored a constant value of E = 210 GPa. The measurements were made using the method of analytical calculation of the eps deformation value from the INT values recorded by the meter. The MagStress meter was calibrated on s960QL steel samples with dimensions of 200 × 30 × 10 mm. During the calibration procedure, the calibration sample was bent with 4-point bending in the range of deformation ε from −0.7 × 10^−3^ to 0.7 × 10^−3^ while measuring strain both by the Barkhausen method and the strain gauge which was taken as a reference value.

### 2.7. Quantitative Phase Analysis and Stress Measurements Using X-Ray Diffraction

X-ray examination of the analysed materials was performed using a Panalytical X’Pert PRO device (Almelo, The Nerherlands) with filtered cobalt anode tube radiation (λ = 1.79021 A). Qualitative X-ray phase analysis of the tested materials was performed in a Bragg–Brentano system using an Panalytical Xcelerator strip detector (Almelo, The Nerherlands). Based on the assumption that the intensity of radiation diffracted on the crystallographic planes of the alpha and gamma phase components was proportional to the share of these phases in the tested materials, the volume share of austenite was estimated using the method proposed by Averbach and Cohen. The direct comparison method based on the above assumption required the measurement of the total intensity of two or more diffraction maxima of the alpha and gamma phases, with the alpha phase serving as a reference for austenite. In this study, it was assumed that the chemical composition of the alpha phase is the same as that of the gamma phase [37,38,39]. The measurement of stresses in the analysed materials using the XRD method was performed using the sin 2ψ technique based on the proprietary X’Pert Stress Plus software v1.9a, which contains a database of material constants necessary for the calculations. In the sin 2ψ method, based on the effect of diffraction line shifts for different ψ angles occurring under stress conditions in materials with a crystalline structure, a silicon strip detector was used on the deflected beam side. The angles of inclination of the samples, ψ, relative to the primary beam were varied in the range of 0° ÷ 75°. The analysis was performed in a direction parallel to the welding direction based on the analysis of the Fe (211) reflection. The positions of the recorded reflections were determined using the Gaussian curve fitting method.

## 3. Result and Discussion

### 3.1. Welding Technology Qualification Test Results

The welded joints were subjected to a welding qualification test procedure in accordance with ISO 15614-1:2017. This standard states acceptance criteria for S960QL steel joints as follows: min. tensile strength R_m_ of 980 N/mm^2^, no inconsistencies over 3 mm after bending with 90 mm radius former, min. Charpy V impact strength of 40 J/cm at −20 °C, max. hardness of 450 HV10 without PWHT and 380 HV10 after PWHT. The destructive testing results are shown in Table 4. The bending test shows no cracks and tears above the acceptance criterion. The impact test yielded results above the min. acceptable impact strength. The highest Vickers hardness with a load of 10kgf was observed for joints after peening, but the results fulfilled the acceptance criteria.

### 3.2. Joint Microstructure Analysis

**Figure 1:**
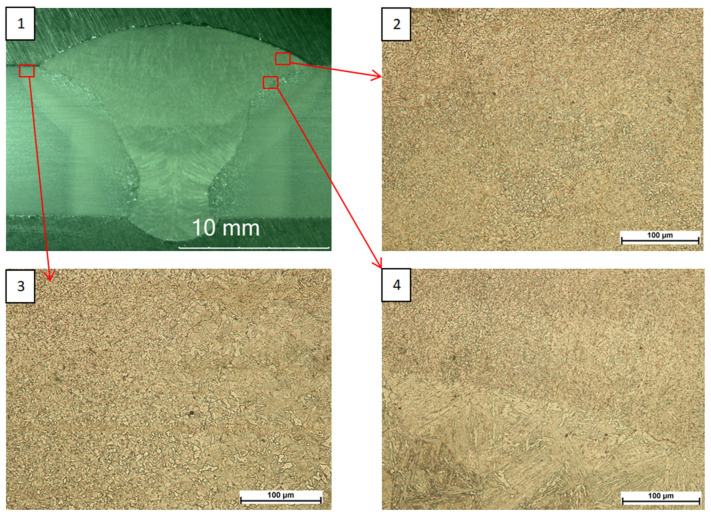
S960QL as-welded joint macrostructure (**1**), face microstructure (**2**), HAZ microstructure (**3**), fusion line microstructure (**4**).

**Figure 2:**
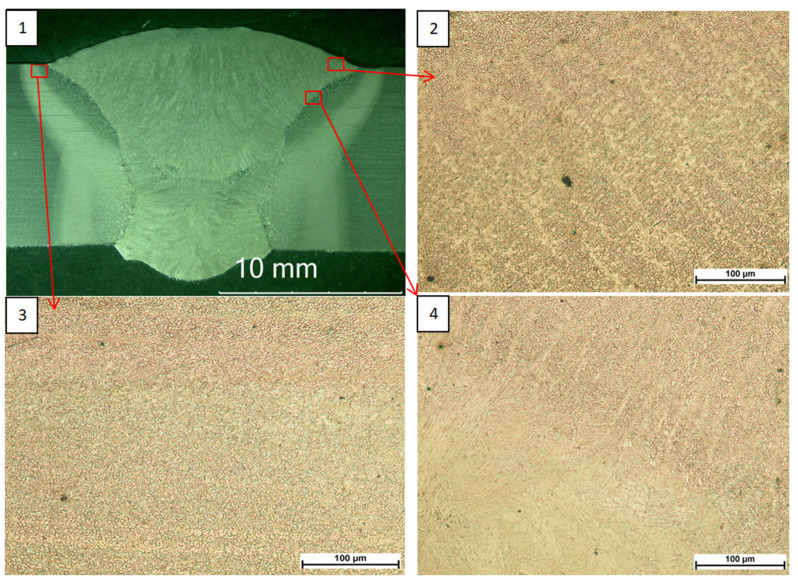
S960QL peened joint macrostructure (**1**), face microstructure (**2**), HAZ microstructure (**3**), fusion line microstructure (**4**).

**Figure 3:**
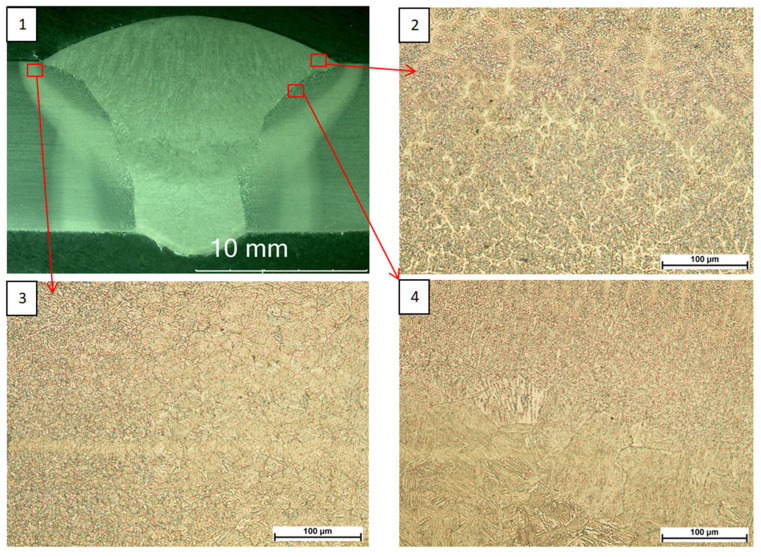
S960QL PWHT joint macrostructure (**1**), face microstructure (**2**), HAZ microstructure (**3**), fusion line microstructure (**4**).

Observations made with a light microscope (Figure 1, Figure 2 and Figure 3) did not reveal any unacceptable inconsistencies in the cross-section of the welded joints. In the case of joints subjected to high-frequency peening, a smoother transition zone from the parent material to the weld face was observed due to the process used.

**Figure 4:**
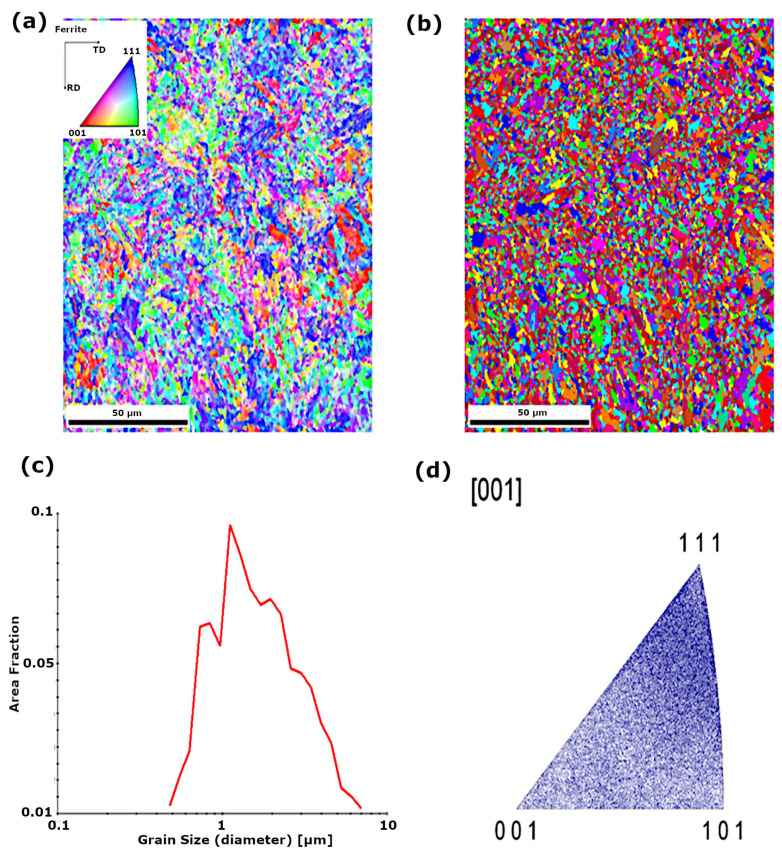
EBSD structural analysis of S960QL steel in the parent material area: (**a**) IPF map of ferrite grain crystallographic orientation, (**b**) grain map, (**c**) grain size distribution, (**d**) inverse pole figure for the [0 0 1] direction—ferrite.

**Figure 5:**
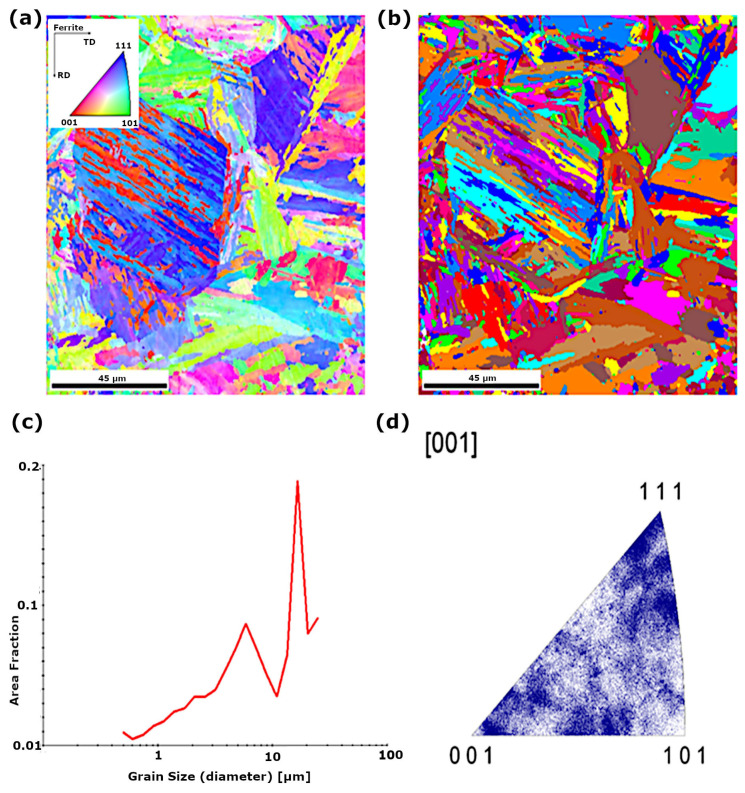
EBSD structural analysis of S960QL as-welded joint fusion zone: (**a**) IPF map of ferrite grain crystallographic orientation, (**b**) grain map, (**c**) grain size distribution, (**d**) inverse pole figure for the [0 0 1] direction—ferrite.

**Figure 6:**
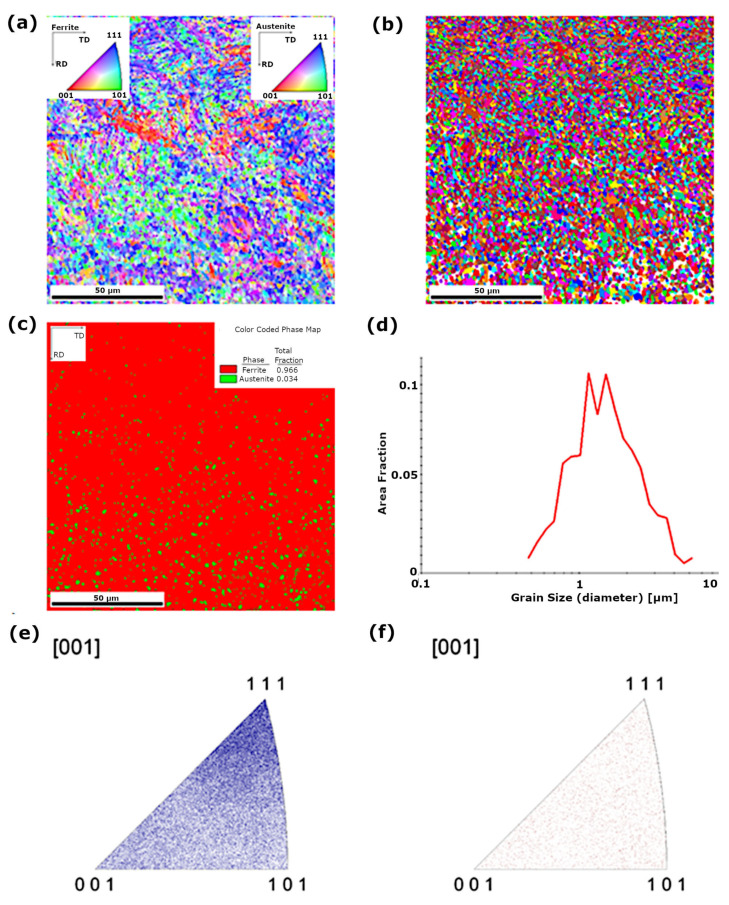
EBSD structural analysis of S960QL peened joint fusion zone: (**a**) IPF map of ferrite grain crystallographic orientation, (**b**) grain map, (**c**) ferrite and austenite phase map, (**d**) grain size distribution, (**e**) inverse pole figure for the [0 0 1] direction—ferrite, (**f**) ) inverse pole figure for the [0 0 1] direction—austenite.

Structural analysis using backscattered electron diffraction (EBSD) in the area of the parent material of S960QL steel showed that the base material is characterised by a ferritic matrix with a grain size in the range of approximately 1–10 µm (Figure 4c). In the case of the welded joint in the as-welded condition in the fusion line and HAZ, ferrite grain growth to a size in the range of approximately 5–30 µm was observed (Figure 5c). Based on the analysis of the inverse pole figures (Figure 5d), a texture was identified in the structure of the fusion line and HAZ. In the case of the joint subjected to peening in the HAZ and fusion line area, both ferrite with a grain size in the range of approximately 0.7–1.5 µm and a few austenite grains were found (Figure 6d). The quantitative share of ferrite is approximately 96.6%, and that of austenite approximately 3.4% (Figure 6c). Based on the analysis of the inverse pole figure results (Figure 6), no preferred grain orientation was observed in the structure of the parent material and in the HAZ.

### 3.3. Phase Composition Analysis Using X-Ray Diffraction

**Figure 7:**
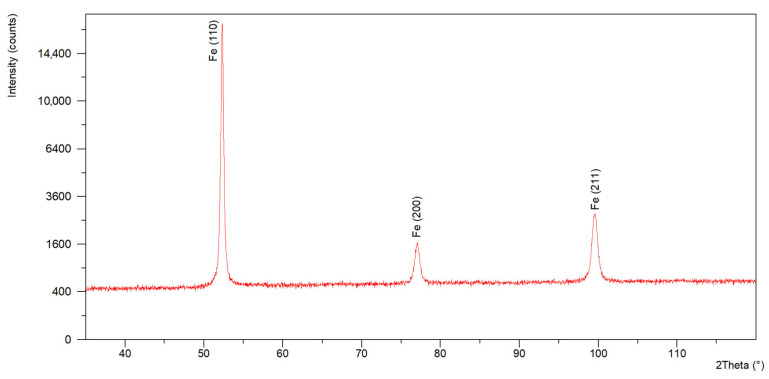
Diffractogram recorded for the parent material.

**Figure 8:**
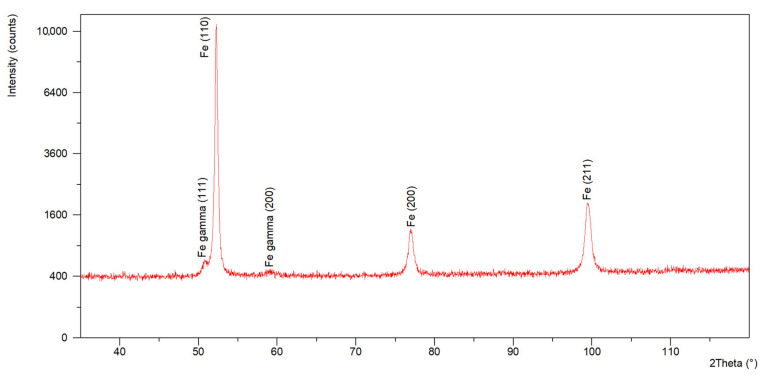
Diffractogram recorded for the fusion zone.

X-ray diffraction (XRD) tests conducted using a diffractometer generating Co Kα radiation passing through a filter (λ = 1.79021 A) confirmed the presence of diffraction reflections (diffraction lines) originating from the family of crystallographic planes (110), (200) and (211) characteristic of ferrite (Figure 7). The results obtained for the weld material, in addition to the presence of diffraction reflections (diffraction lines) originating from the family of crystallographic planes (110), (200) and (211) characteristic of ferrite, also confirmed the presence of a family of crystallographic planes (111) and (111) and (200) characteristic of Fe-γ austenite (Figure 8). The volume fraction of austenite was approximately 4%. The presence of austenite in the welds was due to the nickel content in the welding filler material and the stabilisation of the austenitic phase.

### 3.4. Stress Measurement Analysis

**Figure 9:**
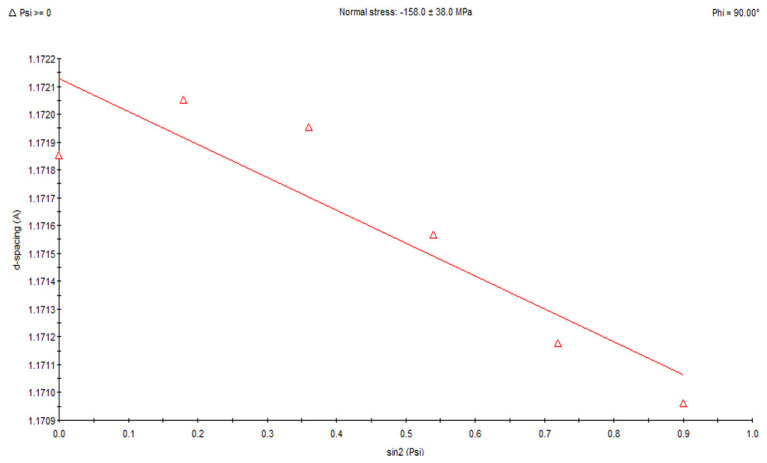
Changes in the interplane distance, d, of the (211) reflection as a function of sin 2ψ, determined for the weld fusion zone after peening.

**Figure 10:**
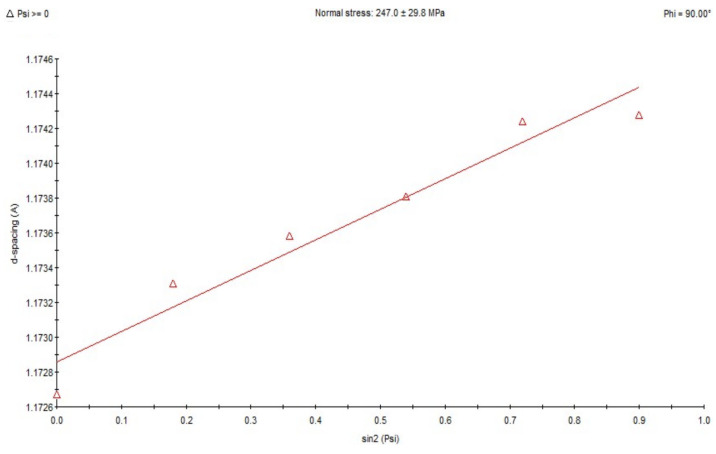
Changes in the interplane distance, d, of the (211) reflection as a function of sin 2ψ, determined for the weld fusion zone in the as-welded state.

**Figure 11:**
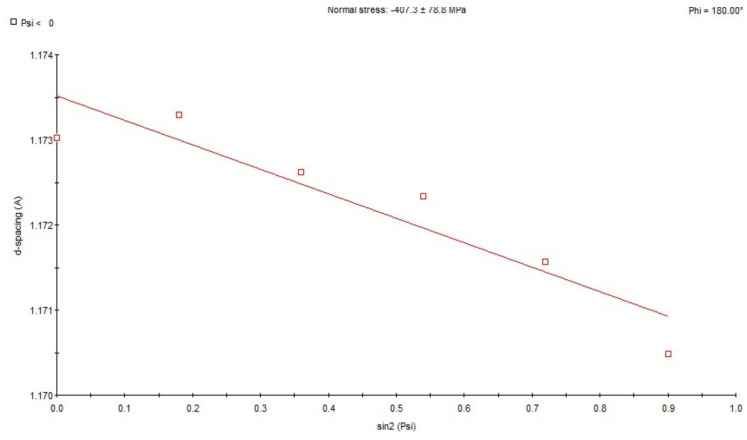
Changes in the interplane distance, d, of the (211) reflection as a function of sin 2ψ, determined for HAZ adjacent to the fusion line after peening.

**Figure 12:**
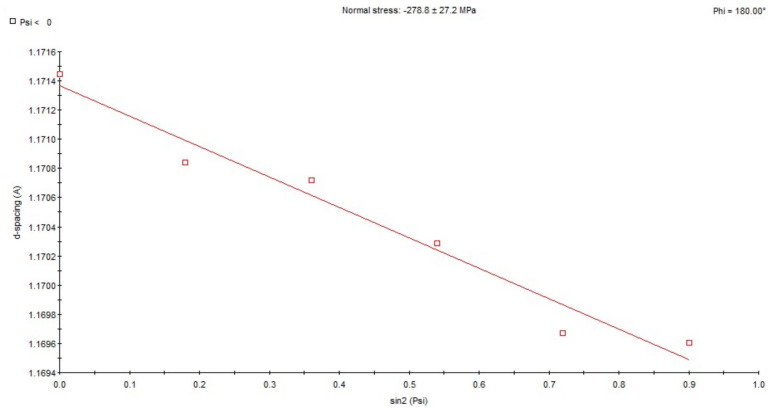
Changes in the interplane distance, d, of the (211) reflection as a function of sin 2ψ, determined for the HAZ adjacent to the fusion line in the as-welded state.

X-ray analysis using the sin 2ψ method showed that in the area subjected to high-frequency peening, compressive stresses ranging from approx. −158 MPa to −206 MPa occur in the direction of welding (Figure 9). In samples taken from the weld in its post-welding state without any additional treatment, tensile stresses ranging from approx. +85 MPa to +247 MPa are present (Figure 10). For the HAZ in the area of the fusion line after peening, compressive stresses of approx. −407 MPa occur (Figure 11), while for the same area of the fusion line of the joint in the as-welded condition, compressive stresses of approx. −278 MPa occur (Figure 12).

**Figure 13:**
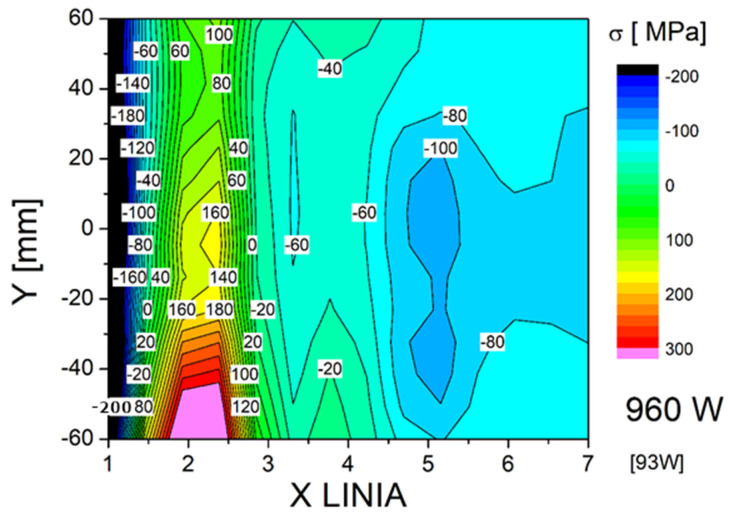
Map of stress distribution in the surface layer of the joint in the as-welded state.

**Figure 14:**
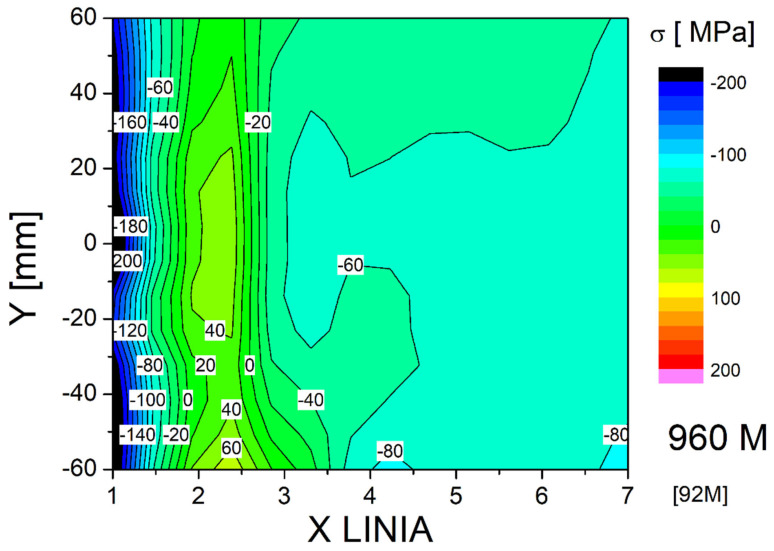
Map of stress distribution in the surface layer of the joint after peening.

**Figure 15:**
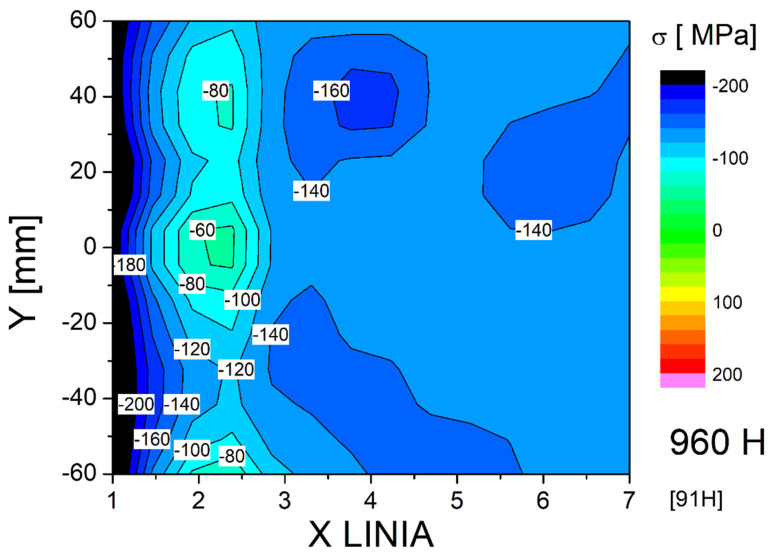
Map of stress distribution in the surface layer of the joint after PWHT.

In addition to XRD analysis, stress measurements were also performed using the Barkhausen effect with a MagStress5c device. The average measurement results are presented in Table 5. The stress results obtained depending on the process conditions and the test area were compiled in the form of 2D maps (Figure 13, Figure 14 and Figure 15). Stress distribution in the welded joints was influenced by the base and additional materials, welding method and parameters, joint and bevel geometry, welded part geometry, and temperature and cooling parameters during welding. In all of the test plates, the fusion zone surface was characterized b a significant level of compressive stresses. Compressive stresses in the surface of a welded joint are beneficial in dynamic loading or fatigue scenarios. The high mean and maximum tensile stresses observed in the as-welded joint HAZ can promote cracking. The coarse-grained HAZ area was determined by Y. Bai et al. as one of the fatigue-cracking-susceptible zones in HSLA steel-welded joints. Both tested treatments reduced the tensile stresses, with PWHT introducing compressive stresses to the HAZ surface. The base material surface was characterized by compressive stresses after welding with applied treatments increasing the level measurably. The characteristics of the stresses combined with the base material structure obtained during the steel making process showed that this zone should not impact joint properties. PWHT proved to be much more effective in improving the surface HAZ stress level and distribution. However, PWHT was much more expensive than HFMI treatment and introduces additional problems to welding technology [40,41,42].

## 4. Conclusions

The tests carried out showed that the high-frequency peening technology used after each stitch did not adversely affect the results of all the required tests during the qualification of the S960QL plate-welding technology in comparison with the test plates in the as-welded and post-stress-relief heat treatment state. Based on the tests performed after welding 10 mm thick S960QL plate metal using the MAG method on a robotic workstation, the following conclusions can be drawn:

➢ Transverse tensile testing of all three test plates showed tensile strength, Rm, results were over the required value of 980 N/mm^2^;
➢ Transverse bending tests on samples with face tension and edge tension showed no non-conformities and met the ISO 15614-1:2017 requirements;
➢ Impact testing of test plates in the as-welded state and with inter-stitch peening yielded similar results, and in the case of samples taken from the welded joints in the post-heat treatment state, the test results from the heat-affected zone were on average about 30J higher than for the other two plates. This may be due to a decrease in hardness in the HAZ as a result of stress relief annealing, which was confirmed during the test.
➢ The hardness test of the welded joint in the as-welded condition in the parent material area showed an average value of 356 HV10; for the fusion zone, it was 345 HV10, and for the heat-affected zone, it was 315 HV10. In the case of the test plate after stress relief annealing, the values were as follows: parent material—357 HV10, fusion zone—365 HV10, and HAZ—307 HV10. In the case of the test plate after peening, the values were as follows: parent material—352 HV10, fusion zone—337 HV10, and HAZ—328 HV10. There is a noticeable increase in hardness in the HAZ area that has been peened.
➢ Observation with a light microscope showed that in the HAZ area directly below the plate surface, the microstructure is fragmented, which is most likely caused by intense plastic deformation during the HFMI process and the resulting deformation. The observation of the microstructure under a light microscope was supplemented by EBSD analysis, which confirmed differences in grain size in the zone directly below the surface.
➢ Phase composition analysis using X-ray diffraction, as well as EBSD analysis, showed that the fusion zone contains approximately 4% Fe-γ austenite by volume. This is due to the increased nickel content in the electrode wire used to weld these samples (2.24% by weight).
➢ Both tested treatments resulted in significant improvement in HAZ stresses, with PWHT achieving better improvement. Both of the tested treatments did not negatively impact mechanical properties in any significant manner. While PWHT looks like a more promising technology, it possesses significant downsides vs. HFMI, mainly a higher cost.

## Figures and Tables

**Table 1:** Chemical composition of welded base material S960QL.

Plate	Element Concentration, wt%													
	C	Mn	Si	P	S	Cr	Mo	Ni	V	Cu	Al	Ti	Nb	As
S960QL	0.12	1.42	0.47	0.008	0.003	0.59	0.56	0.79	0.05	0.11	0.007	0.005	0.02	0.002

**Table 2:** Chemical composition of ED-FK 1000 wire.

Wire	Element Concentration, wt%											
	C	Mn	Si	P	S	Cr	Ni	V	Cu	Al	Mo	Ti + Zr
ED-FK	0.11	1.75	0.81	0.007	0.009	0.36	2.24	0.002	0.009	0.005	0.57	0.08

**Table 3:** Welding parameters of test joints.

Sample Designation	Bead No	Interpass Temperature [°C]	Average Welding Current [A]	Average Arc Voltage [V]	Welding Time [min]	Linear Energy [kJ/mm]
S960QL as welded	1	28.6	201	23.9	2:35	1.07
	2	82.5	203	27.0	1:27	0.70
	3	104.5	255	26.3	2:42	1.35
S960QL + HMFI	1	47.5	200	23.8	2:38	1.04
	2	89.6	202	27.1	1:29	0.71
	3	114.5	255	26.3	2:31	1.34
S960QL + PWHT	1	36.0	202	24.0	2:51	1.06
	2	92.4	202	27.0	1:27	0.71
	3	119.7	254	26.4	2:29	1.37

**Table 4:** Destructive testing results.

Test Type	Sample	Result						Sample						Result					
**S960QL as welded**																			
Transverse tensile test	TT-1	1007 MPa						TT-2						1011 MPa					
Transverse bending tests	TFBB1	positive						TRBB1						positive					
	TFBB2	positive						TRBB2						positive					
Charpy impact testing	VWT 0/2	58.9 J						VHT 0/2						82.4 J					
	VWT 0/2	54.9J						VHT 0/2						79.5 J					
	VWT 0/2	59.8 J						VHT 0/2						74.6 J					
Hardness testing	Ma-1 HV10	L1	367	370	356	313	301		335	341	331	330	352		285	334	365	353	357
		L2	350	351	345	279	290		334	366	349	356	349		316	279	340	359	356
**S960QL peened**																			
Transverse tensile test	TT-1	1011 MPa						TT-2						989 MPa					
Transverse bending tests	TFBB1	positive						TRBB1						positive					
	TFBB2	positive						TRBB2						positive					
Charpy impact testing	VWT 0/2	58.9 J						VHT 0/2						66.7 J					
	VWT 0/2	58.9 J						VHT 0/2						70.6 J					
	VWT 0/2	58.9 J						VHT 0/2						68.7 J					
Hardness testing	Ma-1 HV10	L1	355	366	362	303	360		326	326	370	361	365		385	327	315	355	360
		L2	356	341	334	282	278		356	336	320	309	326		334	295	316	353	356
**S960QL PWHT**																			
Transverse tensile test	TT-1	998 MPa						TT-2						992 MPa					
Transverse bending tests	TFBB1	positive						TRBB1						positive					
	TFBB2	positive						TRBB2						positive					
Charpy impact testing	VWT 0/2	52.0 J						VHT 0/2						117.7 J					
	VWT 0/2	96.1 J						VHT 0/2						116.7 J					
	VWT 0/2	101.0 J						VHT 0/2						100.0 J					
Hardness testing	Ma-1 HV10	L1	360	361	370	300	319		328	334	355	349	307		290	310	338	344	347
		L2	356	354	344	292	288		345	371	387	398	330		300	294	327	329	344

**Table 5:** Summary of mean stress values measured using the Barkhausen method.

	Mean Stress [MPa]		
Test Sample	Fusion Zone	HAZ	Parent Material
S960QL as welded	−170	+180	−40
S960QL peened	−150	+30	−65
S960QL PWHT	−170	−90	−140

## Data Availability

The original contributions presented in this study are included in the article. Further inquiries can be directed to the corresponding authors.
